# Sample‐to‐cutoff ratios using Architect HIV Ag/Ab Combo: The influence with the results of supplemental tests and optimal cutoff value to predict HIV infection

**DOI:** 10.1002/jcla.22866

**Published:** 2019-02-25

**Authors:** Linchuan Wang, Jing‐Yuan Wang, Xu‐Dong Tian, Jin‐xiong Ruan, Yan Yu, Fang Yan

**Affiliations:** ^1^ The First Affiliated Hospital of Xi’an Jiaotong University Xi’an China; ^2^ Hong‐Hui Hospital Xi’an Jiaotong University College of Medicine Xi’an China; ^3^ Xi’an NO.3 Hospital Xi’an China

**Keywords:** HIV, Architect HIV Ag/Ab Combo, sample‐to‐cutoff

## Abstract

**Background:**

The Architect HIV Ag/Ab Combo has excellent performance for HIV screening; however, the false‐positive rate (FPR) was high in low HIV prevalence setting.

**Objectives:**

The purpose of this study was to analyze the influence of sample‐to‐cutoff (s/co) ratios by Architect HIV Ag/Ab Combo with the results of confirmatory test and explore the potential utility of s/co to predict HIV infection.

**Methods:**

A retrospective review on Architect HIV Ag/Ab Combo reactive results was performed at a teaching hospital in Xi'an. The s/co values in different groups, that is, true positives (TP) and false positives (FP), different Western blotting (WB) bands among WB‐positive cases, were compared. The receiver operating characteristic curve (ROC) analysis was used to determine the optimal cutoff value for predicting HIV infection.

**Results:**

During the study period, 219 out of 84 702 patients were reactive by ARCHITECT with a 0.0992% of HIV prevalence and a 56.25% of FPR. The mean s/co ratios in TP were significantly higher than that in FP (458.15 vs 3.11, *P* < 0.0001). Among the WB‐positive cases, the s/co ratios increased significantly with the increase in the number of bands, *P* = 0.0065. The optimal cutoff (24.44) by ROC analysis can provide the highest sum of sensitivity (100%) and specificity (100%) with no FP results.

**Conclusions:**

For Architect HIV Ag/Ab Combo, the FPR is reduced when s/co ratios increase, and the s/co ≥24.44 may be reliable to predict HIV infection.

**Abbreviations**
AIDS: acquired immune deficiency syndrome
CDC: Center for Disease Control and Prevention
FP: false positives
HIV: human immunodeficiency virus
MMT: methadone maintenance treatment
s/co: sample‐to‐cutoff
TP: true positives
WB: Western blotting

## 1 BACKGROUND

HIV/AIDS is a serious public health issue. Data from World Health Organization (WHO) have shown that people living with HIV (PLWH) were more than 35 million and 940 000 people died from HIV‐related causes globally in 2017.^1^ Since the HIV/AIDS was first reported in Beijing and Zhejiang Province in 1985,^2^, ^3^, ^4^ the spread rate of HIV in China is alarming. Quite a few prevention policies and tremendous efforts, such as ban on imported blood products since 1985,^3^ blood donors must be tested for HIV since 1995, methadone maintenance treatment program and four free one care,^2^, ^3^, ^4^, ^5^, ^6^ have been made and performed for HIV/AIDS transmission in China. However, the HIV/AIDS epidemic in China is still not controlled. The registered PLWH in mainland China have increased from 664 751 in 2016 to 758 610 in 2017 and 810 910 at the end of June 2018,^7^ although HIV prevalence remained rather low at an estimation of 0.06%.^8^

The ARCHITECT HIV Ag/Ab Combo, which based on a chemiluminescent immunoassay with the ability to simultaneously detect p24 antigen and HIV‐1/2 antibodies, has shortened the window period by around 1 week compared to the third‐generation assay^9^, ^10^, ^11^ and has a broader dynamic readout range. However, the rate of false positives (FP) by the fourth‐generation assay was high in situations of low HIV prevalence.^12^, ^13^, ^14^ The present study was carried out to analyze the influence of s/co ratios by Architect HIV Ag/Ab Combo with the results of supplemental tests and explore the optimal cutoff value to predict HIV infection.

## 2 METHODS

### 2.1 Study population

This study was conducted between June 2017 and January 2018 at the First Affiliated Hospital of Xi'an Jiaotong University. During the study period, 84 702 patients underwent HIV screening (50 143 males [59.2%]; median age 51 years [range: 1 month‐88 years]). Two hundred and nineteen cases repeatedly reactive to initial test by HIV Ag/Ab Combo were included, 160 males (73.06%) and with a mean age of 44.28 years (range: 1 month‐88 years). The data in the study were available from the LIS of the First Affiliated Hospital of Xi'an Jiaotong University and Xi'an Center for Disease Control and Prevention (CDC), Shaanxi Province, China.

### 2.2 Two‐step HIV diagnostic algorithm

The nucleic acid and p24 antigen tests were not applied at Xi'an CDC; meanwhile, low HIV prevalence remained in China and positive predictive value of the fourth‐generation assay was poor.^15^, ^16^, ^17^, ^18^ Considering that Western blotting (WB) is a confirmatory test, a WB‐negative result is often regarded as exclusion of HIV infection by the patient and clinician in China. The incorrect understand and treatment may lead to serious consequences for the early infections. Since January 16, 2017, a new delivery protocol of blood samples that only reactive to HIV‐1/2 Ab subjects should be conducted and WB has been developed by Xi'an CDC.

Based on this, a two‐step HIV diagnostic algorithm is currently used at our hospital. The first step, a fourth‐generation kit, Architect HIV Ag/Ab Combo (Abbott Diagnostics, Abbott Park, IL), which allows for identification of acute infection and importantly reduces the antibody‐free “window” period, is used as the HIV‐1/2 initially screening test. The second step, a third‐generation EIA kit, XinChuang HIV‐1/2 Ab (InTec, INC, XiaMen, FuJian, China), and a confirmatory test (WB) are used as the supplemental tests for the initially screening reactive cases. For the third‐ and fourth‐generation kits, the s/co ≥1 and s/co <1 were defined as reactive and non‐reactive (negative), respectively.

HIV‐1/2 antibodies and p24 antigen negative should be reported if the initially screening test is non‐reactive. WB should be conducted if it was reactive to HIV‐1/2 Ab. For the only HIV Ag/Ab Combo reactive subjects, the HIV‐1/2 Ab test should be carried out at the 2nd, 4th week, 3rd and 6th month, and the WB should be performed once HIV‐1/2 Ab reactive occurs. For WB‐negative or indeterminate cases, WB should be conducted at the 2nd, 4th week, 3rd and 6th month.

### 2.3 Western blotting

Western blotting (HIV1/2 BLOT 2.2; MP Biomedicals, Singapore) is conducted at the Xi'an CDC, with the separated HIV‐1 gene product groups of *gag* (p17, p24, p39, p55), *pol* (p31, p51, p66), *env* (gp120, gp160, gp41), and HIV‐2‐specific antigen (gp36) immobilized on the membrane. The HIV‐1 interpretations of Xi'an CDC are as follows: positive—the presence of at least two *env *bands plus one *gag* or one *pol *band; indeterminate—reactivity to any of the bands but not compatible with the positive criteria; and negative—the absence of any of the specific bands or the presence of only p17 band. HIV‐2—the presence of gp36 band indicates HIV‐2 infection.

### 2.4 Statistical analysis

Statistical analyses were conducted by SPSS13.0 (serial number 5026743; SPSS Inc, Chicago, IL), and WB positive was the standard for HIV infection diagnosis in the study. The continuous variables were expressed by mean, the Mann‐Whitney *U* test was used between two groups, and multiple groups were performed using the one‐way ANOVA test and Kruskal‐Wallis test. The receiver operating characteristic curve (ROC) analysis was used to determine the optimal cutoff value for predicting true positives (TP). A *P*‐value <0.05 (two‐tailed) was considered as statistically significant.

## 3 RESULTS

### 3.1 The results of two‐step algorithm

**Figure 1:**
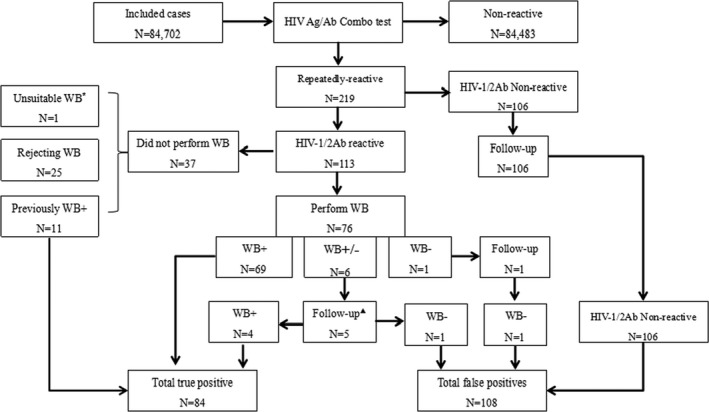
The schematic distributions of sample results

**Figure 2:**
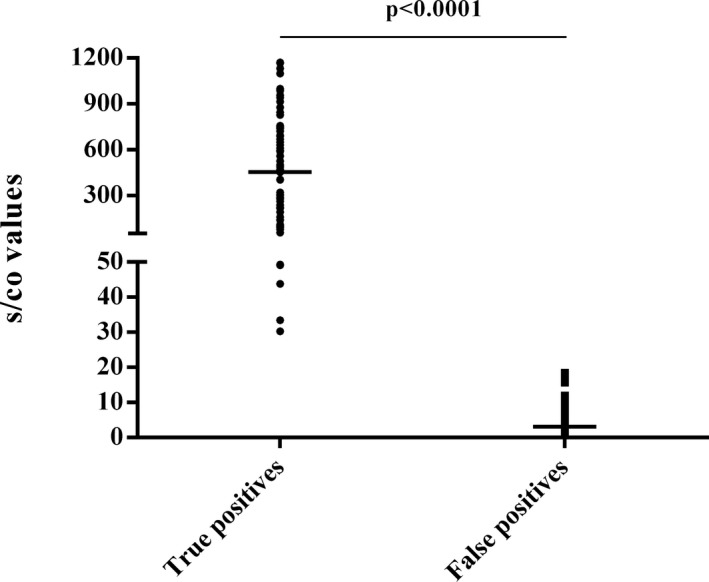
The comparison of s/co values using Architect HIV Ag/Ab Combo in true and false positives

During the study period, 219 out of 84 702 patients were repeatedly reactive by ARCHITECT HIV Ag/Ab Combo. Of these, 113 and 106 were reactive and non‐reactive to HIV‐1/2 Ab EIA, respectively. Among the 113 HIV‐1/2 Ab reactive cases, 11 were previously WB positive, 1 was unsuitable for WB (1 month of age), and 25 rejected WB test. According to the HIV diagnostic algorithm used in our hospital, 76 and 106 cases performed WB test and follow‐up HIV‐1/2 Ab EIA, respectively. Overall, 84 and 108 cases were identified as TP (WB+: 69; follow‐up WB+: 4; previously WB+: 11) and FP (follow‐up WB‐: 2; follow‐up EIA‐: 106), respectively, Figure 1. The mean s/co ratios of HIV Ag/Ab Combo in TP were significantly higher than that in FP (458.15 vs 3.11, *P* < 0.0001), Table 1 and Figure 2.

**Table 1:** The characteristics of HIV Ag/Ab Combo reactive cases

Parameter	Sex			S/CO:Mean (range)	Age: Mean (range)
	Male	Female	Total		
Total no. of reactive to HIV Ag/Ab Combo	160	65	219	157.08 (1‐1171.22)	44.28 (1 mo‐88 y)
No. of non‐reactive to HIV1/2 Ab EIA^a^	48	58	106	3.03 (1‐18.55)	44.64 (2‐88 y)
No. of reactive to HIV1/2 Ab EIA	106	7	113	458.51 (3.65‐1171.22)	38.31 (1 mo‐84 y)
Reactive to Ag/Ab Combo and HIV1/2 Ab EIA					
No. of previously WB positive	10	1	11	163.63 (62.45‐1132.65)	44.80 (28‐55 y)
No. of rejecting WB test	23	2	25	164.45 (27.54‐1068.56)	44.43 (21‐75 y)
No. of unsuitable WB test (≤18 mo of age)	0	1	1	306.18	1 mo
WB test and follow‐up results					
Positive	66	3	69	160.05 (30.32‐1171.22)	44.30 (20‐69 y)
Negative (seroconversion)	1 (0)	0	1 (0)	11.99	24 y
Indeterminate (seroconversion)	6 (4)	0	6 (4)	64.32 (3.65‐99.1)	45.50 (23‐84 y)
Overall^b^	130	62	192	202.19 (1‐1171.22)	45.71 (2‐88 y)
True positives	80	4	84	458.15 (30.32‐1171.22 )	39.27 (20‐69 y)
False positives	50	58	108	3.11 (1 −18.55)	50.72 (2‐88 y)

a Seroconversion was not observed during follow‐up period.

b “Rejecting WB test,” “unsuitable WB test,” and “lost to follow‐up” cases were excluded.

### 3.2 Analysis of Western blotting results

Among the 69 WB‐positive cases, there were 15 kinds of WB patterns, 84.06% (58/69) cases presented with reactivity to more than or equal to seven antigens patterns, and the s/co ratios had a statistically significant association with the band numbers, *P* = 0.0065. According to the follow‐up results of the WB‐negative and indeterminate cases (n = 7), a WB‐negative case (s/co = 11.99) and a WB indeterminate case (s/co = 3.65, p24 band) were confirmed as negative. However, four WB indeterminate cases (s/co: 39.1‐91.4) had seroconversion, and a WB indeterminate case with p24 gp160 bands (s/co = 93.24) was lost to follow‐up, Table 2. Overall, a total of 73 cases were confirmed as WB positive. Among them, on the basis of the criteria of laboratory stages of HIV infection by Fiebig et al,^18^ 4, 8, and 61 cases can be classified as stage IV (EIA+ and WB indeterminate), V (EIA+, WB+ but lacking p31) and VI (EIA+, WB+ and presence p31), respectively, Table 2.

**Table 2:** Analysis of Western blotting results

WB positive (n = 69)				
Bands	WB patterns	No.	S/CO: Mean	Kruskal‐Wallis test
				*P*‐value
10 (All）	p17 p24 p55 p31 p39 p51 p66 gp41 gp120 gp160	23	605.9	0.0065
9	p17 p24 p55 p31 p51 p66 gp41gp120 gp160	19	516.9	
8	p17 p24 p31 p51 p66 gp41 gp120 gp160	2	500.5	
	p17 p24 p55 p51 p66 gp41 gp120 gp160	1		
7	p24 p31 p51 p66 gp41 gp120 gp160	13	397.1	
6	p24 p31 p66 gp41gp120 gp160	1	217.5	
	p24 p51 p66 gp41gp120 gp160	1		
	p17 p24 p66 gp41gp120 gp160	1		
	p31 p51 p66 gp41gp120 gp160	1		
5	p24 p31 gp41gp120 gp160	1	114	
	p17 p24 gp41gp120 gp160	1		
	p24 p31 p66 gp120 gp160	1		
4	p24 p66 gp120 gp160	1	82.2	
	p24 gp41gp120 gp160	1		
3	p24 gp41gp160	2	51.6	

Follow‐up results of WB negative and indeterminate (n = 7)		
WB results	WB patterns (S/CO)	Follow‐up results
Negative	None (11.99)	Negative
Indeterminate	p24 gp160 (49.15)	p24 p51 p66 gp41 gp120 gp160
Indeterminate	p24 gp160 (49.35)	p17 p24 p55 p31 p39 p51 p66 gp41 gp120 gp160
Indeterminate	p24 (91.4)	p17 p24 p55 p31 p39 p51 p66 gp41 gp120 gp160
Indeterminate	gp120 gp160 (39.1)	p24 gp41 gp120 gp160
Indeterminate	p24 (3.65)	Negative
Indeterminate	p24 gp160 (93.24)	Lost to follow‐up

### 3.3 ROC analysis

With the exclusions of certain cases, that is, rejecting WB test (n = 25), unsuitable WB test (n = 1), and lost to follow‐up (n = 1), a total of 84 675 cases were included with a 0.0992% (84/84 675) of HIV prevalence. ROC analysis showed that the optimal cutoff value based on the highest sum of sensitivity (100%) and specificity (100%) was estimated to be 24.44. When the s/co ratios of 1, 9.64 and 17.42 were used as the cutoff value for HIV infection diagnosis, it can achieve 100% of sensitivity and 99.87%‐99.96% of specificity, and false‐positive rate (FPR) decreased from 56.25% to 3.45%. When the cutoff was revised to 31.90 and 38.66, it can achieve 100% of specificity and provide no false‐positive results; however, the sensitivity was decreased to 98.81% and 97.62%, respectively, Table 3.

**Table 3:** Sensitivity and specificity at different cutoff

Parameter	Value(s) based on different cutoff					
	1.00	9.64	17.42	24.44	31.90	38.66
No. of greater than or equal to cutoff^a^	192	91	87	84	83	82
No. of false‐positive results	108	7	3	0	0	0
No. of true HIV‐positive results	73	73	73	73	72	71
No. of previously confirmed positives	11	11	11	11	11	11
Sensitivity (%)	100.00	100.00	100.00	100.00	98.81	97.62
Specificity (%)	99.87	99.991	99.996	100.00	100.00	100.00
False‐positive rate (%)	56.25	7.69	3.45	0.00	0.00	0.00

a “Rejecting WB test,” “unsuitable WB test,” and “lost to follow‐up” cases were excluded.

## 4 DISCUSSION

There is no cure for HIV infection, and the new and recent HIV infections are more infectious than chronic infections.^15^ Thus, the early identifying HIV infection and effective antiretroviral therapy (ART) are key approaches to prevent HIV transmission and control the virus. The Global Health Sector Strategies on HIV (2016‐2021) have proposed that 90% of PLWH can be tested, 90% people diagnosed should be treated, and 90% ART receivers should be virally suppressed in 2020, and the AIDS epidemic as a public health threat should be ended by 2030.^16^ China is low HIV prevalence; however, only 68% of PLWH were diagnosed and 67% of the diagnosed cases received ART in 2015.^8^

Currently in China, the fourth‐generation assay is not widely used to screen HIV due to expensive fees, the nucleic acid (NAT) and p24 antigen tests are also rarely applied at CDC and hospitals for HIV infection diagnosis. The shortcomings result that few of the infections can be detected at the early period and the diagnosis of HIV‐exposed infants <18 months of age has been a challenge. Fiebig et al^17^ classified the laboratory stages of HIV infection as six stages; however, none of the HIV infections in Xi'an can be identified at the stage I (NAT+) and II (NAT and p24 antigen+) because of NAT and p24 antigen tests not applied at CDC. During the study period, 5.48% (4/73), 10.96% (8/73) and 83.56% (61/73) of the new HIV infections can be classified as stage IV, V, and VI, respectively. Based on the result of Fiebig et al,^17^ this hinted that 83.56% of the cases have been infected with HIV more than 2 months.

The “5 C principles” recommended by WHO, that is, informed consent, confidentiality, counseling, correct test results and connection must be followed for all HIV testing services, and the right to decline testing should be recognized. In our study, excluding those previously confirmed HIV‐positive cases, 25 of 102 HIV1/2 Ab reactive subjects (24.51%) declined WB; however, high s/co ratios (range: 27.54‐1068.56) by HIV Ag/Ab Combo were observed. Meanwhile, a 1‐month‐old infant (s/co = 306.18) in the study cannot conduct WB until 18 months due to the NAT not applied at CDC. Another WB indeterminate case (s/co = 93.24, p24, and gp160 bands) was lost to follow‐up. Among the 102 cases who should perform WB, the numbers that HIV‐Ab cannot be confirmed summed up to 27 (26.47%).

The Architect HIV Ag/Ab Combo has proven to be highly reliable for HIV screening; however, the frequency of false‐positive results was high in low HIV prevalence setting.^12^, ^13^, ^14^, ^18^, ^19^, ^20^ In our study population, Architect HIV Ag/Ab Combo can provide high sensitivity (100%) and specificity (99.87%) with a with a 0.0992% of HIV prevalence. However, high FPR (56.25%) was observed, which was similar with other reports in China (39.5%)^18^ and Korea (69.8%).^12^ The mean s/co ratios in TP were significantly higher than that in FP (458.15 vs 3.11). In WB positives, with the increase in the number of bands, the s/co ratios increased significantly. The optimal cutoff value of Architect HIV Ag/Ab Combo in the study, which can provide the highest sum of sensitivity (100%) and specificity (100%) with no false‐positive results, was 24.44 compared with 8.8 in Kim et al,^12^ 11.26 in Cui et al,^18^ and 32.7 in Chacón et al.^14^

There are several limitations in this study. Firstly, 26.47% of the HIV Ag/Ab Combo reactive cases with certain conditions, such as rejecting WB test, unsuitable WB test and lost to follow‐up, could not been identified the status of HIV infection, which may affect the results of the present study. Secondly, the HIV diagnostic algorithm used in the study is based on that the NAT and p24 antigen tests are also rarely applied at CDC and hospitals for HIV infection diagnosis in China, which is not applicate to other regions out of China.

## 5 CONCLUSIONS

In summary, few of the HIV infections detected at the early period and quite a few PLWH unaware their status may be the key factors for the AIDS epidemic uncontrolled or slowed down in China. For HIV Ag/Ab Combo, the s/co ratios are associated with the results of supplementary tests and the s/co ≥24.44 may be reliable to predict the HIV TP.

## AUTHORS’ CONTRIBUTIONS

LW and YY were major contributors in the writing of the article. LW, JYW, and FF were responsible for the study design. The statistical analysis and figure of the study were performed by LW, JYW, JXR, XDT, and YY.

## ETHICS STATEMENT

The study was deemed exempt from review by the Ethics Committee of the First Affiliated Hospital of Xi'an Jiaotong University as routine data for clinical purpose were used and all the information of patients was kept confidential in the study.

## CONSENT TO PARTICIPATE

Not applicable.

## CONSENT FOR PUBLICATION

LW, JYW, JXR, XDT, YY, and FF have read and approved the final work for publication in Clinica Chimica Acta.

## AVAILABILITY OF DATA AND MATERIAL

The data used in the study were available from Xi'an CDC and LIS of the First Affiliated Hospital of Xi'an Jiaotong University.
